# Reheated Palm Oil Consumption and Risk of Atherosclerosis: Evidence at Ultrastructural Level

**DOI:** 10.1155/2012/828170

**Published:** 2012-12-19

**Authors:** Tan Kai Xian, Noor Azzizah Omar, Low Wen Ying, Aniza Hamzah, Santhana Raj, Kamsiah Jaarin, Faizah Othman, Farida Hussan

**Affiliations:** ^1^Department of Anatomy, Faculty of Medicine, Universiti Kebangsaan Malaysia, Jalan Raja Muda Abd Aziz, 50300 Kuala Lumpur, Malaysia; ^2^Institute of Medical Research, Ministry of Health, Jalan Raja Muda Abd Aziz, 50300 Kuala Lumpur, Malaysia; ^3^Department of Pharmacology, Faculty of Medicine, Universiti Kebangsaan Malaysia, Jalan Raja Muda Abd Aziz, 50300 Kuala Lumpur, Malaysia

## Abstract

*Background*. Palm oil is commonly consumed in Asia. Repeatedly heating the oil is very common during food processing. *Aim*. This study is aimed to report on the risk of atherosclerosis due to the reheated oil consumption. *Material and Methods*. Twenty four male Sprague Dawley rats were divided into control, fresh-oil, 5 times heated-oil and 10 times heated-oil feeding groups. Heated palm oil was prepared by frying sweet potato at 180°C for 10 minutes. The ground standard rat chows were fortified with the heated oils and fed it to the rats for six months. *Results*. Tunica intima thickness in aorta was significantly increased in 10 times heated-oil feeding group (*P* < 0.05), revealing a huge atherosclerotic plaque with central necrosis projecting into the vessel lumen. Repeatedly heated oil feeding groups also revealed atherosclerotic changes including mononuclear cells infiltration, thickened subendothelial layer, disrupted internal elastic lamina and smooth muscle cells fragmentation in tunica media of the aorta. *Conclusion*. The usage of repeated heated oil is the predisposing factor of atherosclerosis leading to cardiovascular diseases. It is advisable to avoid the consumption of repeatedly heated palm oil.

## 1. Introduction

Palm oil is one of the commonly consumed oils in Malaysia. It contains 50% unsaturated fat and 50% saturated fat [1]. Consumption of saturated fat is believed to predispose cardiovascular disease. However, fresh palm oil contains tocopherols and tocotrienols which are antioxidants and the antioxidant effect of tocotrienols is 40–60 times higher than that of tocopherols [2]. Frying is one of the common methods to prepare Asian food. Therefore, the consumed fats in our diet are exposed to extreme temperature during cooking. Furthermore, the practice of reusing oils for repeated frying is also prevalent in an attempt to save cost. Repeated heating increases lipid peroxidation and reduces antioxidant properties of the oils, leading to produce free radicals [3]. Moreover, free radical induced oxidative stress is associated with the atherosclerosis development [4]. Therefore, the ingestion of repeatedly heated oil might produce harmful effect, attributing to the development of atherosclerosis.

Atherosclerosis is a chronic progressive disease which commonly affects arteries, resulting in reduced blood flow that eventually predisposes to various ailments such as coronary artery disease and cerebrovascular disease. Atherosclerosis is prevalent in all over the world. It has been proven that nutrition and cholesterol intake in the diet are ranked as the highest risk factors in atherosclerosis [5]. The incidence of the disease is usually associated with the circulating low-density lipoprotein (LDL). Polyunsaturated fatty acid residues in lipoprotein are vulnerable to free radical oxidation and these modified oxidative LDLs are scavenged by the macrophage, forming the cholesterol laden “foam-cells” in the atherosclerotic plaques [4]. Furthermore, these LDLs are chemoattractants for macrophage and smooth muscle cells [6]. The atheromatous plaques formation might be related to the growth factor action and angiogenesis property of vasoactive small molecules produced by the mast cells [7]. Therefore, cholesterol laden macrophage, mononuclear cells migration, and migratory smooth muscle cells in tunica media are expected to reveal in atherosclerotic plague at ultrastructural level. The present study aimed to highlight the impact on the cardiovascular health by consumption of reheated palm oil and review the literature on the mechanism of development of atherosclerosis.

## 2. Materials and Methods

The aortic samples of this study were obtained from the previous research conducted by the postgraduate student of the Department of Pharmacology, Faculty of Medicine, UKM [8]. The protocol of the study was as follows.

### 2.1. Experimental Animals

Twenty-four healthy adult male Sprague-Dawley rats (200–280 g) were obtained from the institutional animal resource unit. The rats were reared in stainless steel cages with a room temperature of 27 ± 2°C with 12 hours light and dark cycle. All rats were allowed to access food and tap water *ad libitum*. All the animals handling procedures were in accordance with the institutional animal ethical guideline with the ethical approval number UKMAEC: FP/FAR/2008/Kamsiah/9-Apr/220-Apr-2008-Feb-2011.

### 2.2. Source and Preparation of Diets

Commercial palm oil (Lam Soon Edible Oil, Malaysia) was used as fresh, five times heated and ten times heated as described by Owu et al. [9]. Briefly, the 2.5 L of the oil was used to fry 1 kg of sweet potatoes in a stainless-steel wok at 180°C for 10 minutes. To prepare five times and ten times heated oil, the hot oil was allowed five hour cooling interval, and the entire frying process is then repeated four and nine more times, respectively, with a fresh batch of sweet potatoes. No fresh oil was added between batches to replace any loss due to evaporation and absorption of oil. The test diets were formulated by mixing 15% weight/weight of the respective prepared oils with ground standard rat chow (Gold Coin Sdn Bhd, Malaysia), reformed into pellets, and then dried in an oven overnight at 70°C. The preparation of test diet was in accordance with the experimental protocol of Adam et al. [10]. However, cholesterol was not added in the present study.

### 2.3. Study Design

This study was a randomized control study. The rats were acclimatised for one week prior to feed the test diets. They were randomly divided into 4 groups of six based on the diet, namely, basal diet feeding group (C), basal diet fortified with 15% weight/weight fresh palm oil (FPO), 5 times heated palm oil (5HPO), and 10 times heated palm oil (10HPO) feeding groups. After 6 months of feeding with the respective diets, all the rats were sacrificed using diethyl ether. The proximal portion of the ascending aorta and the arch of aorta were taken for light and electron microscopic studies.

### 2.4. Sample Preparation

Each aortic sample was sectioned into 3 segments of less than 1.0 mm thickness. They were initially immersed for 12–16 hours at 4°C in glutaraldehyde fixative. The samples were then washed 3 times in 0.1 M phosphate buffer, bulk stained with 1% buffered osmium tetroxide for 1-2 hours, and washed in distilled water for 3 times. They were then treated with uranyl acetate for 30 minutes, dehydrated in an ascending series of ethanol solution, infiltrated in propylene oxide, and finally embedded in resin at 60°C for 24 hours. After the resin had polymerized, the samples were sectioned with glass knives.

### 2.5. Histomorphometric Study

Semithin sections of 1 *μ*m thickness with 1% toluidine blue staining were viewed using a computerized image analyzer of 100 times magnification with the software Image-Pro Plus (Version 5.0.2.9, Media Cybernetics, Inc., Bethesda, USA) together with light microscope (Eclipse 80i, Nikon Corporation, Tokyo, Japan). The aortic section was nominally divided into 4 quarters, and the tunica intima and tunica media thickness were measured at 5 different random areas for each quarter. The mean of the 20 readings was then taken as a representative of a particular treatment group and used for statistical analysis. Thickened tunica intima in the sample was selected for qualitative electron microscopic study.

### 2.6. Qualitative Electron Microscopic Study

The resin blocks were further trimmed at the areas of interest (thickened tunica intima) to identify the ultrastructural changes. Ultrathin sections of 80 nm thickness were then collected and stained with 3% uranyl acetate and Reynold's lead citrate. These specimens were examined with a transmission electron microscope (Philips HMG 400, Philips, Eindhoven, The Netherlands) for the presence of vacuoles and mononuclear cells in the tunica intima. Micrographs were then taken for qualitative description.

### 2.7. Statistical Analysis

The data was presented as the mean ± standard error of mean (SEM). Normally distributed data were analysed using parametric tests analysis of variance (ANOVA) test. Data that were not normally distributed were analysed using nonparametric tests, Mann-Whitney *U* test. Results were considered significant if the *P* value is <0.05. All mentioned statistical analyses were conducted using Statistical Product and Service Solutions (SPSS) software, version 13.

## 3. Results

### 3.1. Quantitative Analysis

The quantitative data were shown in Table 1. The analysis of all the measurements was done by comparing between the frequencies of heating.

#### 3.1.1. Tunica Intimal (TI) Thickness

In general, there was a significant difference in the TI thickness among all groups (*P* = 0.013). However, in terms of types of diets used, there was no significant difference of TI thickness among control and the fresh oil groups (*P* = 0.065).

Comparison among groups of rats fed with palm oil with different frequency of heating (fresh (FPO), 5-times (5HPO) and 10-times heated (10HPO)) was done. Results show that there was a significant difference of TI thickness in the palm oil group (*P* = 0.012), whereby the 10-times heated group has the thickest TI, followed by 5-times heated and lastly, fresh palm oil.

#### 3.1.2. Tunica Media (TM) Thickness

There was no significant difference of TM thickness between control and fresh oil feeding groups as well as among the frequencies of heating and fresh oil feeding groups (*P* > 0.05).

#### 3.1.3. Tunica Intima to Tunica Media Ratio (IMR)

The analysis of IMR was done by comparing between the frequencies of heating and fresh oil. In general, there was a significant difference in the IMR among all groups (*P* = 0.009). In terms of types of diets, there was also a significant difference in IMR between the control and palm oil groups (*P* = 0.019).

**Figure 1:**
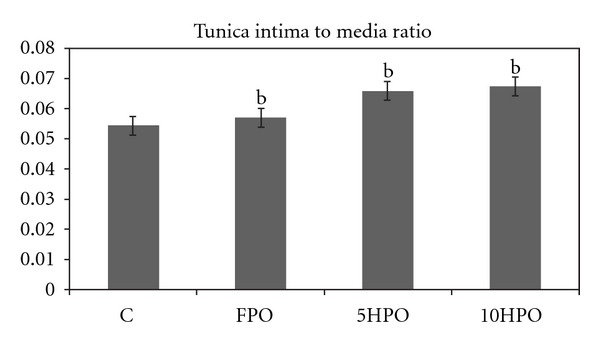
Tunica intima to tunica media ratio (IMR) in the aortic wall of the rats fed with different frequencies of heated palm oil. ^b^Significant difference between control and palm oil fed groups (*P* < 0.05).

Comparison among groups of rats fed with fresh (FPO) and repeatedly heated palm oil (5HPO and 10HPO) showed no significant difference of IMR (*P* = 0.281). However, the IMR among the palm oil groups showed an increasing trend (Figure 1).

### 3.2. Qualitative Analysis

**Figure 2:**
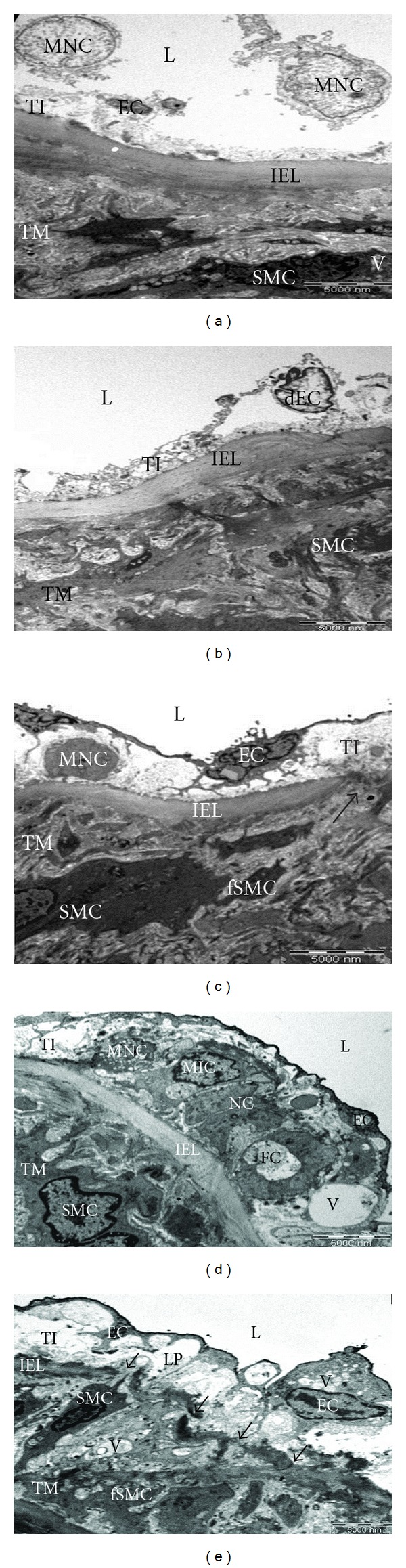
EM 1: Ultrastructure of the aortic wall of rats fed with different frequencies of heated oil (3200x magnification). (a) Control, (b) fresh palm oil (FPO), (c) 5 times heated palm oil (5HPO), and ((d) and (e)) 10 times heated palm oil (10HPO). Tunica intima (TI); tunica medica (TM); vessel lumen (L); denuded endothelial cells (dEC); mononuclear cells (MNC); internal elastic lamina (IEL); smooth muscle cells (SMC); endothelial cell (EC); granular material (GM); fragmented SMC (fSMC); central necrotic core (NC); myointimal cells (MIC); vacuoles (V); foam cells (FC); disrupted IEL (arrows); lipid accumulation (LP).

For qualitative descriptive analysis, the changes in the ultrastructure of aortae of the rats (*n* = 7) were examined using transmission electron microscope. Only one sample from each treatment group which showed the greatest IMR was chosen. The electron micrographs (EM) of the respective groups, as well as their descriptions, are as in Figure 2. The ultrastructure of the aorta of control rat was shown in EM 1 (Figure 2(a)).

The endothelium of the majority of the test diet feeding groups consists of an intact layer of endothelial cells, maintaining their squamous characteristic. In FPO group as shown in EM 1 (Figure 2(b)), the denuded endothelial cells (dEC), with no mononuclear cells (MNC), were found in the TI. The internal elastic lamina (IEL) appeared intact, continuous, and regular and appeared to be normal thickness. The TM consisted mainly of smooth muscle cells (SMC), some of which appeared to be fragmented. No intracellular vacuoles (V) were seen. In 5HPO group as shown in EM 1 (Figure 2(c)), the intact endothelial cell (EC) layer was found as in the FPO. However, there was a thickening with MNC and granular material (GM) in the subendothelial layer. The lipid accumulation and collagenous connective tissue might be the content of GM. There was a gap found in the IEL with migratory features of SMC cytoplasm into the TI was also revealed. The fragmented SMC (fSMC) was found in the TM. However, no intracellular vacuoles were seen. In 10HPO group, the TI appeared to be prominently thickened secondary to the plaque formation. The plaque was characterized by a central necrotic core (NC) with surrounding MNC, myointimal cells (MIC), vacuoles (V), and foam cells (FC) embedded in the granular material and collagenous connective tissue. The lesion projected prominently into the vessel lumen and it was covered by an intact EC layer. The plaque was rested on the IEL which appeared to be intact, continuous, and regular (EM 1 (Figure 2(d)). However, the discontinuous and irregular IEL was found in the other area of the specimen. The fragmented SMC was noted in the TM. The massive lipid accumulation (LP) was also found in the subendothelial layer (EM 1 (Figure 2(e)).

## 4. Discussion

Consumption of saturated fat generally attributes to cardiovascular ailments. Palm oil which is rich in monounsaturated fatty acids is derived from the tropical plant *Elaeis guineensis* [1]. Although it is generally regarded as saturated oil, we must also take into account its antioxidant properties. It contains vitamin E, tocopherols, and tocotrienols which act as potent antioxidants [2]. It helps to protect against lipid peroxidation by trapping free radicals [11]. It has also been proved that the tocotrienol-rich fraction (TRF) in palm oil is known to exhibit cardioprotective effects [12]. Furthermore, the palm oil derived vitamin E possesses the serum lipid lowering properties mainly on cholesterol and low density lipoprotein (LDL) [13].

However, as the antioxidants (vitamin E) are extremely sensitive to heat, the repeated heating reduces the antioxidant properties of oil [3]. Repeated thermal exposure may generate more free radicals in the oil due to the underlying oxidative process [14]. These free radicals are highly reactive to bind with the lipids, proteins, carbohydrates, and DNA in the body system, enhancing oxidative stress [5]. Therefore, consumption of repeatedly heated oil might aggravate the lipid peroxidation, leading to damage the arterial wall and increase uptake of lipid, and, subsequently, develop atherosclerosis [10]. Therefore, the heated oil feeding rats in the present study showed obvious changes in the aortic wall as the oil lost its protective antioxidant properties. The gradually increased TI thickness in the heated oil treated groups indicated that the antioxidant effect of palm oil was gradually lost when reheating frequency was increased. The histomorphometric and morphological findings in our study complimented the fact that heating destroyed the tocotrienols and other heat labile vitamins in the oil, resulting in reduction of antioxidant properties of the oil [3].

As polyunsaturated fatty acid residues in lipoprotein are vulnerable to free radical oxidation, the oxidized LDLs are atherogenic via its cytotoxic effect towards arterial endothelial cells [15]. The oxidatively modified LDLs were found in the human and rabbit atherosclerosis lesion [16]. The modified LDLs induced the transmigration of monocyte into the subendothelial space and it was prevented by pretreatment with antioxidant vitamin E [17]. Moreover, atherosclerotic changes develop when the LDLs infiltrate into the tunica intima and accumulate in macrophages [18]. This event initiates the proliferation of intimal fibroblasts and myointimal cells together with collagen deposition, producing a plaque which causes the intima thickening that is identified as the earliest indicator of atherosclerotic process [19]. The tunica intima thickening in the control group of the present study could be explained by the effect of aging because the duration of study was the total of 6 months. It has been proved that aging is a responsible factor for the differential changes in the TI and TM thickness [20]. However, the intimal thickening in the heated oil treated groups might be the adverse consequence of oil consumption, eventually leading to atherosclerosis.

Furthermore, the increase in IMR indicated the increase in subendothelial ground material accumulation. The slight elevation of IMR in the FPO group was probably due to the fatty acids in nature of the oil although fresh oil possesses antioxidant properties. However, the qualitative results showed no obvious changes in FPO group. The palm oil decreases the serum triglyceride (TG) and cholesterol level [21]. It also increases the level of high density lipoprotein (HDL) [22, 23]. It is well documented that high HDL level may lower the risk of cardiovascular problem [24]. The ultrastructural finding in FPO group of our study implicated the protective role of FPO due to its antioxidant vitamin compositions.

Several studies have been done on consumption of repeatedly heated oil and the impact on cardiovascular diseases. Although one study found the temporary increase in serum TG and LDL level in 5HPO feeding ovariectomised rats, the morphological results showed no obvious changes under light microscopic study [25]. In another study, the ovariectomised rats were fed with cholesterol diet fortified with heated palm oil and they found that the atherosclerotic changes were revealing at the ultrastructural level without significant alteration in the plasma lipid profile [26]. The present study was conducted on the male rats feeding with basal diet without being fortified with cholesterol, yet the ultrastructural level study also revealed the similar finding as in the study done by Adam et al. [26]. This can be concluded as the male rats are more liable to develop cardiovascular diseases.

Several other studies were conducted on the consequence of atherosclerosis which attributes to the aetiology of hypertension and cardiac problem. The study found that the consumption of repeatedly heated oil resulted in high blood pressure [27]. The authors also pointed out that the free radicals generated by repeated heating might impair the nitric oxide bioavailability on the blood vessel wall leading to hypertension [27]. The evidence of atherosclerotic plaque formation in the present study might also contribute the development of hypertension. The necrosis in cardiac tissue was found in repeatedly heated palm oil fed rats [8]. It might be due to atherosclerosis plague formation in the coronary artery, leading to narrowing of the lumen which diameter is much narrower than that of the aorta. The above findings indicated the harmful effect of consumption of reheated oil.

In conclusion, the antioxidant property of oil is reduced by repeated heating that increases the lipid peroxidation which aggravates the development of atherosclerosis. Therefore, it is important that we should utilize protective nutritional value of palm oil in full and discourage the usage of repeated heating oil in our daily diet to reduce the risk of atherosclerosis.

### 4.1. Limitations and Recommendations

Firstly, the development of atherosclerosis in animal models may be different from human, despite the obvious histological similarities of atherosclerosis between both species. Therefore, it is recommended to develop the relevant model to conduct the extended study. 

Secondly, as the selected area of the aortic sample was examined under electron microscope, the certain parts of the aortic sample which would have more relevance to our study might have been missed. In our humble opinion, further detailed and quantitative studies are recommended to explore the exact nature of disease development.

In addition, it would be interesting to investigate the relationship between biochemical parameters and the histomorphometric as well as electron microscopic study in one single research. The future research should be aimed to determine the safe threshold of heating frequency by using the lower frequencies of heating such as one or two times and so on.

## Figures and Tables

**Table 1:** Quantitative analysis of changes in the aortic wall of rats fed with different frequencies of heated palm oil.

Groups	Tunica intima (TI) thickness (*μ*m) ± SEM	Tunica media (TM) thickness (*μ*m) ± SEM	TI : TM ± SEM
Control (C)	23.9200 ± 1.6405	442.5800 ± 18.7733	0.0546 ± 0.0040
Fresh (FPO)	21.6250 ± 1.4244	380.2100 ± 11.9707	0.0570 ± 0.0029^b^
5 times heated (5HPO)	26.7817 ± 1.7856^a^	450.7383 ± 43.6100	0.0659 ± 0.0048^b^
10 times heated (10HPO)	30.1883 ± 2.1573^a^	423.2150 ± 14.8780	0.0673 ± 0.0059^b^

^a^Significant difference between heated oil and fresh oil (*P* < 0.05).

^b^Significant difference between control and palm oil feeding groups (*P* < 0.05).

Results shown as mean ± standard error of mean (SEM).
